# Outcome of untreated lung nodules with histological but no microbiological evidence of tuberculosis

**DOI:** 10.1186/s12879-018-3442-9

**Published:** 2018-10-23

**Authors:** Che-Liang Chung, Yen-Fu Chen, Yen-Ting Lin, Jann-Yuan Wang, Shuenn-Wen Kuo, Jin-Shing Chen

**Affiliations:** 1 Department of Internal Medicine, Yuanlin Christian Hospital, Changhua, Taiwan; 2 Department of Internal Medicine, National Taiwan University Hospital Yunlin Branch, Douliu, Yunlin Taiwan; 3 Department of Internal Medicine, National Taiwan University Hospital, #7, Chung-Shan South Road, Zhongzheng District, Taipei, 10002 Taiwan; 4 Division of Thoracic Surgery, Department of Surgery, National Taiwan University Hospital and National Taiwan University College of Medicine, Taipei, Taiwan

**Keywords:** Caseous necrosis, Granulomatous inflammation, Pulmonary nodule, Surgery, Tuberculosis

## Abstract

**Background:**

The outcome of lung nodule(s) with histopathological findings suggestive of tuberculosis (TB) but lack of microbiologic confirmation remains unclear. Whether these patients require anti-TB treatment remains unknown. The aim of the study was to compare the risk of active TB within 4 years in untreated patients with histological findings but no microbiological evidences suggestive of TB.

**Methods:**

From January 2008 to June 2013, patients with either solitary or multiple lung nodules having histological findings but no microbiological evidences suggestive of TB were identified from a medical center in Taiwan and were followed for 4 years unless they died or developed active TB.

**Results:**

A total of 107 patients were identified. Among them, 54 (51%) were clinical asymptomatic. Biopsy histology showed granulomatous inflammation in 106 (99%), and caseous necrosis was present in 55 (51%) cases. Forty (37%) patients received anti-TB treatment, and 21 (53%) of them had adverse events, including 13 initially asymptomatic patients. Anti-TB treatment was favored in patients with caseous necrosis, whereas observation was preferred in subjects whose nodules were surgically removed. Only 1 case in the untreated group developed culture-confirmed active pulmonary TB during 4-year follow-up (1 case per 251.2 patient-years). None of the 16 cases having co-existing histologic finding of malignancy became incident TB case within a follow-up of 56.7 patient-years.

**Conclusions:**

In patients having lung nodules with only histologic features suggestive of TB, the incidence rate of developing active TB was low. Risk of adverse events and benefit from immediate treatment should be carefully considered.

## Background

Tuberculosis (TB) is an infectious disease prevalent worldwide. Currently, its diagnosis, which is mainly based on medical history, clinical manifestation, radiographic features, microbiological evidence, and laboratory findings, remains challenging. The definite diagnosis of TB is made through a positive culture from infected sputum or tissue samples. In Taiwan, among all infectious diseases, TB is associated with the highest incidence and mortality rate, with 12,338 TB cases (53.0 per 100,000 individuals) recorded in 2012; of these, 19% had sputum smear-negative and culture-negative TB [1]. If noninvasive methods cannot provide a definite diagnosis, presumptive diagnosis can be made through tissue biopsy [2].

The histological features suggestive of TB include granulomatous inflammation, caseous necrosis, and positive acid-fast stain (AFS); however, these are not pathognomonic for active TB. For example, granulomatous inflammation and caseous necrosis may be caused by various pathogens other than *Mycobacterium tuberculosis*, such as nontuberculous mycobacteria (NTM) and fungi [3]. Moreover, granulomatous inflammation may sometimes be seen in noninfectious diseases. The characteristic morphological feature of hypersensitivity pneumonitis is bronchiolocentric granulomatous lymphocytic alveolitis [4]. Sarcoidosis is also characterized by noncaseating granulomas [5].

Solitary or multiple lung nodules are frequently encountered during health checkup and clinical examination. Histological examination of biopsy specimens is often required to establish a definite diagnosis and exclude the possibility of malignancy. However, even if the histological examination reveals findings suggestive of TB, differentiating an active disease from old tuberculoma is difficult without serial radiographic studies. Hence, whether all patients with such nodules should receive standard anti-TB treatment immediately remains unknown.

Therefore, in this study, we identified patients with lung nodules having histological findings but no microbiological evidence suggestive of TB and compared the clinical characteristics and incidence of active TB within the subsequent 2 years between patients receiving and not receiving anti-TB treatment.

## Methods

### Patients and setting

This was a retrospective cohort study conducted in National Taiwan University Hospital (NTUH). The Research Ethics Committee of NTUH approved this study (REC No.: 201205025RIC). Under Taiwan’s National TB control policy, all cases of culture-confirmed and suspected TB should be reported to the Taiwan Centers for Disease Control (CDC), and all TB contacts should receive contact investigation and be reported to Taiwan CDC, as well. Permission is required to access the online reporting database of Taiwan CDC (https://tb.cdc.gov.tw/slow/ca/loginbycard.asp). Standard regimen used in Taiwan for treating new TB cases follows the guidelines of the World Health Organization (WHO), consisting isoniazid (5 mg/kg), rifampicin (10 mg/kg), pyrazinamide (25 mg/kg), and ethambutol (15 mg/kg) for 2 months, followed by isoniazid (5 mg/kg), rifampicin (10 mg/kg), plus ethambutol (15 mg/kg) if results of susceptibility test is not available, for 4 months [6, 7].

We searched the histopathology database of NTUH for findings of caseous necrosis or granulomatous inflammation from January 2008 to June 2013. Patients were excluded if (1) the *M. tuberculosis* complex or NTM were isolated within 60 days before or after biopsy, (2) biopsy samples were extrapulmonary, (3) biopsy was performed during anti-TB treatment, (4) computed tomography (CT) images of the chest were unavailable, (5) histological or microbiological evidence of fungal or parasitic infection was noted, (6) patients had pure or mixed with non-nodular radiographic patterns suggestive for active disease, such as tree-in-buds pattern, consolidation, and miliary lesions [8], and (7) a positive tissue AFS, since these cases were usually treated as TB until proven otherwise.

### Follow-up and outcome

All included patients were followed up for 4 years after biopsy unless they died or received a diagnosis of active TB. Active TB was defined if either of the following two criteria was fulfilled [9, 10]: (1) mycobacterial cultures of sputum or other respiratory samples yielded the *M. tuberculosis* complex; and (2) chest radiography revealed new lesions without other proven etiology, which improved after standard anti-TB treatment, determined by serial chest radiography or by follow-up CT, if the pre-treat pulmonary lesion was not detectable by chest radiography.

### Data collection

Medical records were reviewed to obtain demographic data, including age, sex, symptoms, comorbidity, history of TB, biopsy method, histology, AFS results, mycobacterial culture of sputum and tissue specimens, and adverse events of anti-TB treatment. At the beginning of tissue diagnosis, we obtained laboratory test results, including leukocyte and differential leukocyte counts and hemoglobin, albumin, and C-reactive protein levels. CT images were further reviewed by a pulmonologist. Furthermore, radiographic patterns; presence of solitary or multiple nodules, background fibrocalcified lesions, and bronchiectasis; maximum diameter and density of lesions; cavitation; and mediastinal lymphadenopathy were recorded.

### Statistical analyses

Categorical variables were compared using the *chi*-square test or Fisher’s exact test, as appropriate, whereas continuous variables were compared using independent sample *t* tests. Multivariate logistic regression analysis was performed in a backward manner to identify the predictors for prescribing anti-TB treatment. The analyzed variables included age, sex, history of TB, presence of respiratory or constitutional symptoms suggestive of TB, histological findings (caseous necrosis, granulomatous inflammation on biopsy histology, concomitant malignancy on biopsy histology), biopsy method, mycobacterial culture for respiratory specimens or biopsy specimens, comorbidity (diabetes mellitus, end-stage renal disease, malignancy, hepatitis B virus infection, human immunodeficiency virus infection, organ transplant recipient, alcoholism, and autoimmune disease), findings on chest computed tomography (multiple nodules, lesion size > 3 cm, cavitary lesions, ground-glass opacity, fibrocalcified lesions, mediastinal lymphadenopathy), and laboratory test results (leukocyte count and hypoalbuminemia [defined as serum albumin level < 3.5 g/dL]). Kaplan–Meier curves were generated to represent the time to subsequent development of active TB in different groups and were compared using the log-rank test. All analyses were performed using IBM SPSS statistics (version 23; IBM Corp., Armonk, NY, USA). Statistical significance was set at *p* < 0.05.

## Results

### Case identification

**Fig. 1:**
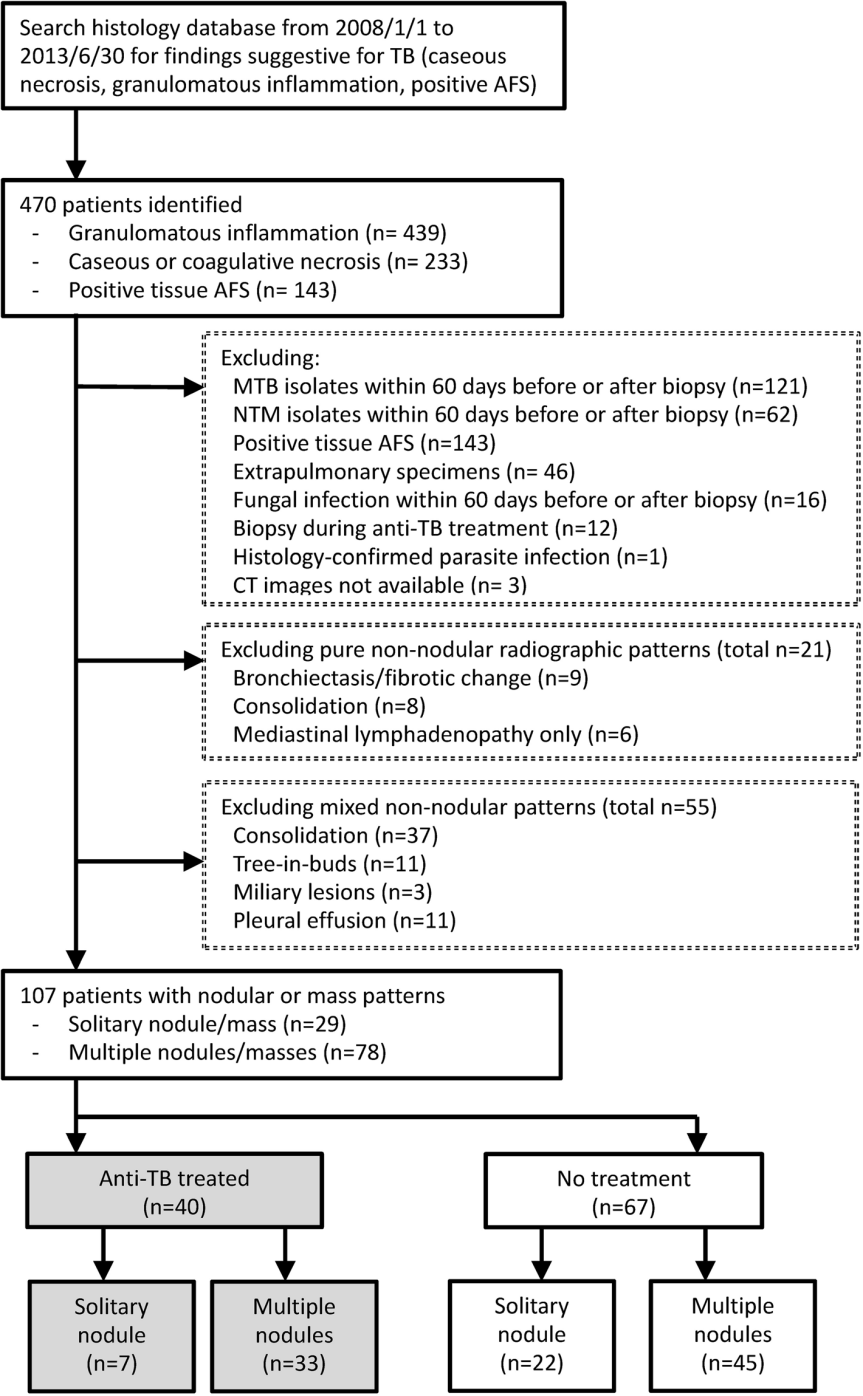
Flowchart of study design and case selection. Selection of patients with lung nodules having histological findings but no microbiological evidence suggestive of tuberculosis

From January 2008 to June 2013, a total of 470 patients with histological findings suggestive of TB were identified; among them, 439 had granulomatous inflammation, 233 had caseous necrosis, and 143 had positive tissue AFS. In total, 363 patients were excluded because of various reasons shown in Fig. 1. The remaining 107 (22.8%) patients were analyzed further.

**Fig. 2:**
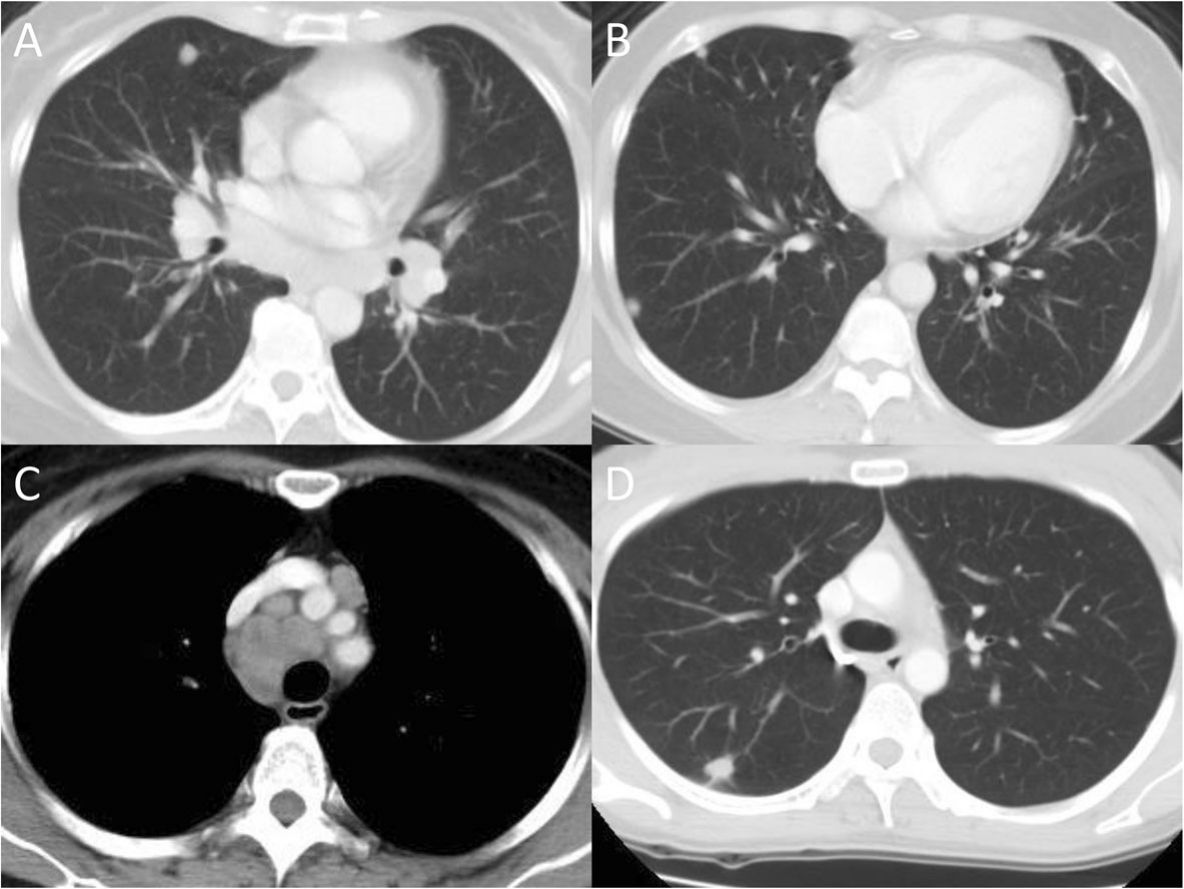
Image presentation of chest computed tomography (CT) in 2 cases. Chest CT of a patient, aged between 50 and 60 (untreated group), revealed multiple round nodules in the bilateral lungs (**a**, **b**) and mediastinal lymphadenopathy (**c**). Chest CT of a patient, aged between 30 and 40 (treated group), revealed an ill-defined speculated, 1.2-cm nodule with pleural tagging at the posterior aspect of the right upper lobe (**d**)

Among all 107 patients, 54 (51%) were clinically asymptomatic and were referred for further evaluation because of abnormal chest images noted during health examination or routine checkup for other medical problems. Among all 107 patients, 40 (37%) received anti-TB treatment (treated group); of them, 17 were clinically asymptomatic. The remaining 67 patients (63%) received follow-up without immediate treatment (untreated group). Among all 107 cases, 78 had multiple lung nodules or masses, while the remaining 29 patients had solitary nodule. Typical CT images of solitary and multiple lung nodules or masses are illustrated in Fig. 2.

### Clinical characteristics and follow-up

The clinical characteristics of all 107 patients are presented in Table 1. The mean age was 56.3 ± 13.4 years, with a male predominance (60%). The most common underlying disease was malignancy (35%), followed by diabetes mellitus (10%). Tissue samples were collected through surgical resection in 65 patients (61%). In total, 91 patients (85%) were tested through sputum or tissue mycobacterial culture. The most common histological finding was granulomatous inflammation (99%), followed by caseous necrosis (51%); 18 patients (17%) had concomitant histological findings of malignancy.

**Table 1:** Clinical characteristics, radiographic patterns, and laboratory data of treated and untreated groups

	All patients (*n* = 107)	Treated group (*n* = 40)	Untreated group (*n* = 67)	*P* value
Age (year)	56.3 ± 13.4	55.5 ± 12.4	56.8 ± 14.0	0.629
Male gender	64 (60)	26 (65)	38 (57)	0.398
Clinically asymptomatic	54 (51)	17 (43)	37 (55)	0.203
Previous history of tuberculosis				0.272
No	95 (89)	38 (95)	57 (85)	
Yes, treatment status unknown	1 (1)	0	1 (2)	
Yes, treated	11 (10)	2 (5)	9 (13)	
Biopsy method				0.090
Bronchoscopy	6 (6)	1 (3)	5 (8)	
CT-guided	33 (31)	18 (45)	15 (22)	
Echo-guided	3 (3)	1 (3)	2 (3)	
Surgery	65 (61)	20 (50)	45 (67)	
Histology				
Granulomatous inflammation	106 (99)	40 (100)	66 (99)	> 0.999
Caseous necrosis	55 (51)	31 (78)	24 (36)	< 0.001
Lymph node sampling	32 (30)	3 (8)	29 (43)	< 0.001
Lymph node involvement	13 (12)	2 (5)	11 (16)	0.125
Concomitant malignancy	18 (17)	2 (5)	16 (24)	0.012
Mycobacterial culture				
Tissue culture performed	68 (64)	21 (53)	47 (70)	0.066
Sputum culture performed	81 (76)	33 (83)	48 (72)	0.205
Either one	91 (85)	34 (85)	57 (85)	0.992
Comorbidity				
Malignancy	37 (35)	6 (15)	31 (46)	0.001
Diabetes mellitus	11 (10)	2 (5)	9 (13)	0.204
ESRD under regular hemodialysis	2 (2)	1 (3)	1 (2)	> 0.999
Liver cirrhosis	2 (2)	2 (5)	0	0.138
Organ transplant recipient	2 (2)	1 (3)	1 (2)	> 0.999
Autoimmune disease	2 (2)	0	2 (3)	0.527
Hepatitis B virus infection	8 (8)	3 (8)	5 (8)	> 0.999
HIV infection	2 (2)	2 (5)	0	0.138
Alcoholism	1 (1)	1 (3)	0	0.374
Main findings on chest CT				
Multiple nodules	78 (73)	33 (83)	45 (67)	0.084
Solitary nodule	29 (27)	7 (18)	22 (33)	0.084
Lesion size > 3 cm	9 (8)	2 (5)	7 (10)	0.400
Associate findings^a^				
Cavitation	3 (3)	0	3 (5)	0.291
Ground glass opacity	20 (19)	9 (23)	11 (16)	0.435
Calcification	22 (21)	9 (23)	13 (19)	0.701
Fibrosis	36 (34)	15 (38)	21 (31)	0.514
Bronchiectasis	13 (12)	5 (13)	8 (12)	> 0.999
Mediastinal LAP	72 (67)	25 (63)	47 (70)	0.415
Lab data				
Albumin (g/dL)^b^	4.2 ± 0.6	4.4 ± 0.5	4.1 ± 0.6	0.005
Hemoglobin (mg/dL)	13.0 ± 1.8	13.4 ± 1.6	12.8 ± 1.9	0.091
Leukocyte count (K/uL)	7.2 ± 3.4	7.2 ± 4.1	7.2 ± 3.0	0.968
Segment (%)	62.4 ± 10.2	61.5 ± 9.9	62.9 ± 10.4	0.479
Band (%)	0.05 ± 0.30	0.10 ± 0.47	0.02 ± 0.12	0.293
Lymphocyte (%)	29.0 ± 9.2	30.8 ± 8.8	27.9 ± 9.2	0.120
CRP (mg/dL)^c^	3.1 ± 5.5	2.8 ± 3.2	3.2 ± 5.9	0.824
Death during follow up period	11 (10)	3 (8)	8 (12)	0.464
Mean follow-up duration (days)	1395.3 ± 217.0	1440.4 ± 85.5	1368.4 ± 263.4	0.097
Developing active tuberculosis	1 (1)	0	1 (2)	> 0.999

*CRP* C-reactive protein, *CT* Computed tomography, *ESRD* End-stage renal disease, *HIV* Human immunodeficiency virus, *LAP* Lymphadenopathy, Data are expressed as number (%) or mean ± standard deviation

^a^Each case might have more than one associate finding

^b^Data were missing for 2 cases of the treated group

^c^Data were available for 9 and 35 cases of the treated and untreated groups, respectively

The mean follow-up duration was similar between the treated and untreated groups. During the follow-up period, 3 treated and 8 untreated patients died. Of the 8 untreated patients, 3 received surgical resection of the lesion. Five died of malignancy, and the remaining 3 died of heart disease, upper gastrointestinal bleeding, and pneumonia, respectively. Half of the 8 untreated patients had the past history of treated pulmonary TB; seven of them (88%) had been tested through either sputum or tissue mycobacterium culture, or both. No evidence of active TB was noted during the entire clinical course of the 8 cases.

No significant difference was observed in age, sex, clinical symptoms, and history of TB between the treated and untreated groups. More patients in the untreated group had malignancy than did those in the treated group (46% vs. 15%, *p* = 0.001). More patients in the treated group had the histological features of caseous necrosis (78% vs. 36%, *p* < 0.001), but fewer patients had the histological features of concomitant malignancy (5% vs. 24%, *p* = 0.012). Anti-TB treatment was not administered to 24 of the 55 patients (44%) with biopsy histology showing caseous necrosis; none of these patients developed active TB in the subsequent 4 years.

The radiographic patterns and laboratory data of the patients are summarized in Table 1. In general, most patients had normal hemogram and serum albumin levels. The laboratory data were similar in the treated and untreated groups, except that the treated group had higher albumin levels. In both groups, the most common radiographic findings were multiple lung nodules and mediastinal lymphadenopathy. The associated findings, such as cavitary lesions and ground-glass opacity, did not differ significantly between the 2 groups. Fibrocalcified lesions, bronchiectasis, and mediastinal lymphadenopathy, implying inactive or chronic disease status, did not differ significantly between the 2 groups.

### Predictors of anti-TB treatment initiation

The multivariate logistic regression analysis identified 2 independent factors for predicting whether physicians initiate anti-TB treatment: caseous necrosis on biopsy histology (OR = 9.60, 95% CI = 3.38–27.23), and surgical resection (OR = 0.25, 95% CI = 0.09–0.69; Table 2).

**Table 2:** Factors associated with prescribing anti-tuberculosis treatment in logistic regression analysis

	Adjusted Odds Ratio^a^	95% CI	*p* Value
Surgical resection	0.25	0.09–0.69	0.007
Caseous necrosis on biopsy histology	9.60	3.38–27.23	< 0.001

*CI* Confidence interval

^a^Adjusted variables included age, sex, history of TB, symptomatic, granulomatous inflammation on biopsy histology, concomitant malignancy on biopsy histology, mycobacterial culture for respiratory specimens or biopsy specimens, comorbidity (diabetes mellitus, end-stage renal disease, malignancy, hepatitis B virus infection, human immunodeficiency virus infection, organ transplant recipient, alcoholism, and autoimmune disease), findings on chest computed tomography (multiple nodules, lesion size > 3 cm, cavitary lesions, ground-glass opacity, fibrocalcified lesions, mediastinal lymphadenopathy), leukocyte count, and hypoalbuminemia

### Development of active pulmonary TB

In the untreated group, during a total of 251.2 follow-up patient-years, only 1 patient developed pulmonary TB approximately 19 months after biopsy (Day 574). None of the 16 cases having co-existing histologic finding of malignancy became incident TB case within a follow-up of 56.7 patient-years.

**Fig. 3:**
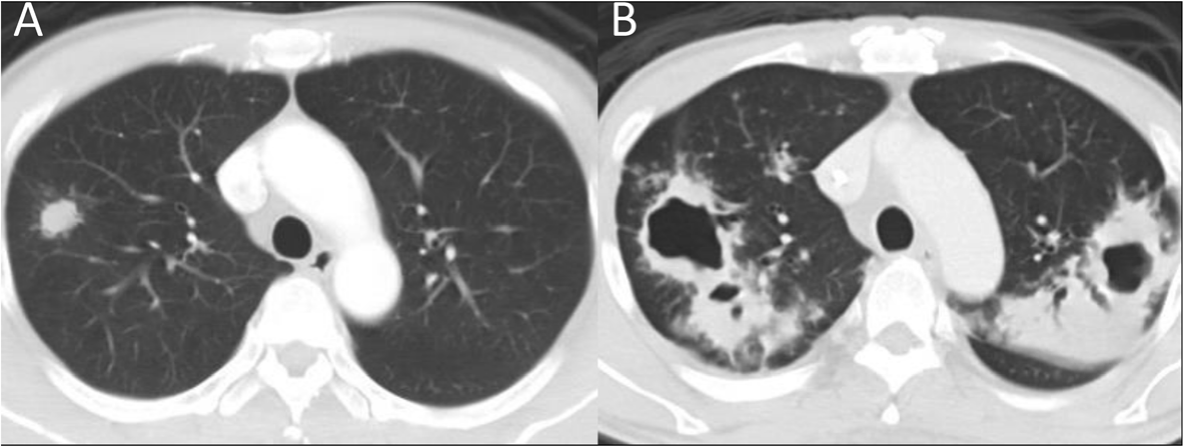
Chest CT of an asymptomatic patient, aged between 50 and 60 (untreated group), revealed a 1.8-cm nodule in the right upper lobe (**a**). Follow-up chest CT at 17 months later showed multiple bilateral cavitary consolidations (**b**). Sputum culture yielded the *Mycobacterium tuberculosis* complex

The patient, aged between 50 and 60, had a history of left maxillary acinic cell carcinoma, which had been surgically resected 13 years ago and had recurred 2 months before the index biopsy. Chest CT for staging work-up revealed a 1.8-cm nodule in the right upper lobe (Fig. 3a). The case reported weight loss and a tendency to become easily fatigued, but denied any airway symptoms. CT-guided biopsy revealed granulomatous inflammation with lymphocyte aggregation and negative tissue AFS. Sputum AFS and mycobacterial cultures were also negative. Anticancer chemotherapy and target therapy were administered consequently. Although follow-up chest CT at 10 months after the index biopsy showed progression in bilateral alveolar infiltrates, sputum studies for a total of 6 specimens collected at 9 and 11 months after the index biopsy were all negative for the *M. tuberculosis* complex. Follow-up chest CT at 17 months after the index biopsy demonstrated multiple bilateral cavitary consolidations (Fig. 3b). Sputum culture at that time yielded the *M. tuberculosis* complex. No records of recent contact to active TB case were found in the online reporting database of Taiwan CDC.

Among the 40 treated patients, none fulfilled the criteria of active TB in the 4 years after the date of the index biopsy. The risk of active TB did not differ between the treated and untreated groups (*p* = 0.433; power = 0.118, Kaplan–Meier analysis).

### Adverse events of anti-TB treatment

**Fig. 4:**
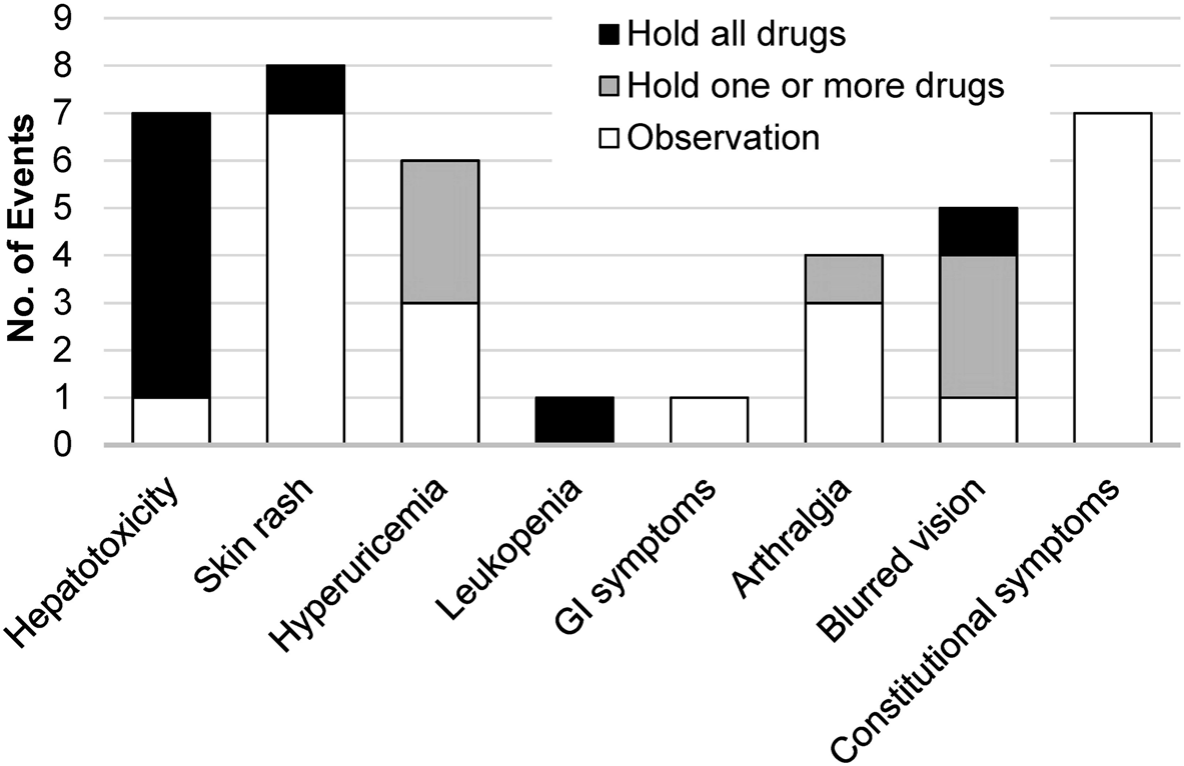
Summary of adverse events caused by anti-tuberculosis treatment in the treated group

Among the 40 patients receiving anti-TB treatment after the index biopsy, adverse events occurred in 21 (53%). The most common adverse event was rash (20%), followed by hepatotoxicity (18%) and constitutional symptoms including fever, malaise, dizziness, and chest tightness (18%; Fig. 4). The development of adverse events led to treatment interruption in 8 patients (20%), including 6 patients who developed hepatotoxicity. Among the 17 clinically asymptomatic patients who received anti-TB treatment, 13 (76%) had adverse events, leading to therapeutic regimen modification in 3 patients (18%) and treatment interruption in 6 patients (35%).

## Discussion

This is the first study to investigate the incidence of active TB within the subsequent 4 years in patients with histological findings but no microbiological evidence suggestive of TB. This study has 3 major findings. First, if untreated, the incidence of active TB was 1 in 251.2 patient-years, which represents an annual incidence of 398 cases per 100,000 individuals (approximately 7 times the incidence in Taiwan); immediately anti-TB treatment may not be necessary in most of these cases. Second, if treated, the risk of adverse events was 53%; one-fifth of the treated cases even demonstrated severe adverse events necessitating interruption of their anti-TB treatment. Third, primary physicians appeared to favor anti-TB treatment in patients with caseous necrosis but appeared to disfavor it in those whose lung nodules had been surgically resected.

The understanding of the natural course of pulmonary tuberculoma is mainly based on reports before the establishment of effective anti-TB treatment. Approximately 30–50% of the cases of pulmonary tuberculoma may demonstrate a stationary course [11–13]. In an early report recording 18 patients with 23 tuberculomas totally, 11 tuberculomas calcified or showed no interval changes during the follow-up period of 12–148 months, and the remaining 12 tuberculomas showed progressive changes within 5–90 months [12]. Currently, anti-TB treatment is typically administered postoperatively when tuberculoma is diagnosed [13–15].

The histological examination of tissue sections of mycobacterial lesions often shows few or no acid-fast bacilli, even when the culture result is positive. This might result from the effects of the fixation fluid or organic solvent [16]. In a report, the identification of mycobacteria through Ziehl–Neelsen staining was accurate in only approximately 60% of culture-positive cases [17]. Some reports have indicated that the nucleic acid amplification (NAA) test for *M. tuberculosis* complex is much more sensitive than the AFS histopathological test; however, the NAA test cannot differentiate between live and dead TB bacilli [16, 18].

The proportion of NTM recovered from AFS-positive respiratory specimens was highly variable among the geographic areas and populations, ranging from 7.3 to 50%, which reduced the positive predictive value of AFS for pulmonary TB [19–22]. In a study on 360 biopsy specimens, mycobacterial culture of 166 specimens was performed. Of them, the percentages of specimens that were culture-positive for mycobacteria with histological features of necrotizing granulomas, nonnecrotizing granulomas, and poorly formed granulomas were 38.2%, 32.4%, and 30.0%, respectively. Of the 39 specimens that were culture-positive for mycobacteria, 14 (36%) yielded the *M. tuberculosis* complex, whereas the remaining 25 (64%) yielded NTM [23]. In a retrospective study, the “TB-like” granulomatous reaction (epithelioid cell granuloma with central caseous necrosis) was the most common pathological feature in NTM infections [24].

In addition to *Mycobacteria*, granulomatous inflammation can be seen in infections caused by fungi, parasites, and *Actinomycetes* [17, 25]. However, the occurrence of active TB in all patients with histological features of caseous necrosis or granulomatous inflammation varies because of the differences in local prevalence. Thus, no histological finding has been identified to be pathognomonic for active TB. Therefore, whether immediately anti-TB treatment should be administered to patients with histological findings but no microbiological evidence suggestive of TB remains debatable [25, 26]. Our study demonstrated that primary care physicians favored anti-TB treatment for patients with histological findings of caseous necrosis. However, no untreated patient with similar histological features of caseous necrosis developed active TB within the subsequent 4 years.

Eighteen (17%) cases in this cohort had concomitant findings of malignancy in biopsy specimens. There were no incident TB cases in these patients, while one TB case was noted during a follow-up of 194.5 patient-years for those without co-existing malignancy in biopsy samples. The prevalence of coexistence of granulomatous inflammation and malignancy in lung biopsy is unknown. Previous reports showed coexisting malignancy in 3 of 616 lung specimens with granulomatous inflammation [27–29]. Because of retrospective design, the reasons for the primary care physicians not favoring anti-TB treatment in patients with underlying malignancy are unknown. These patients might be so weak that anti-TB drugs were considered too toxic as compared with close observation. On the contrary, some doctors might start treatment immediately because of the fear of disseminated TB during subsequent cancer chemotherapy.

Surgical procedures to reduce the bacterial load or to collapse the lung are the mainstay treatments during the preantibiotic period [13, 30, 31]. Because of the high surgical complication rate [14] and development of effective treatment, surgery is currently only indicated as a diagnostic procedure, an adjuvant for multidrug-resistant TB, and a therapeutic strategy for refractory lesions despite adequate anti-TB treatment [13, 32]. Theoretically, if pulmonary lesions are removed completely, the remaining tissues are macroscopically and microscopically normal. In this case, a patient can be considered to be either cured or having latent TB infection (LTBI), which carries the lifetime risk of subsequent active TB by 5–15% [33].

With the increasing use of chest CT for health checkups, the identification of lung nodules has increased [34]. Therefore, pulmonary lesions with histological findings but no microbiological evidence suggestive of TB have become frequently encountered clinical problems. However, guidelines for managing this special clinical entity have not been well established. Given that in our cohort study, none of the 45 untreated patients who received surgical resection developed active pulmonary TB during 4-year follow-up, and only 1 patient in the whole untreated group became incident case, the TB incidence rate in this special group maybe lower than that in LTBI cases [34]. Another concern for initiating empirical anti-TB treatment is the frequently encountered adverse events, particularly in patients with systemic comorbidity, such as malignancy, hypoalbuminemia, and anemia [35–37]. Considering the low risk of progression into active TB and the high risk of adverse events, the decision on either regular follow-up or immediate initiation of anti-TB treatment should be carefully judged based on risk and benefit assessment. A prospective study is required to confirm this finding and our speculation.

This study has some limitations. First, although demographic data and radiological findings were not significantly different between the treated and untreated groups, the physician’s judgment on initiating anti-TB treatment may still bias the risk of subsequent active TB (confounded by indication). Second, while NAA test [38] and immunohistological staining [39] for *Mycobacterium tuberculosis* complex on biopsy specimens are promising for the diagnosis of active TB, they were not routinely performed in all hospitals in Taiwan and most hospitals in the world. Finally, in some cases, respiratory specimens for AFS and mycobacterial culture were not collected because of the lack of sputum; however, this might not be a major concern and might indicate no active pulmonary disease.

## Conclusion

In patients having lung nodules with histological findings but no microbiological evidence suggestive of TB, the incidence rate of active TB is 1 in 251.2 patient-years, lower than that in LTBI cases. Given the low risk of active TB and the high risk of adverse events, regular follow-up sputum and imaging studies, rather than immediate anti-TB treatment administration, may be considered. However, an additional prospective study is necessary to confirm our current findings.

## Abbreviations

AFS: Acid-fast stain
CI: Confidence interval
CT: Computed tomography
LTBI: Latent tuberculosis infection
NAA: Nucleic acid amplification
NTM: Nontuberculous mycobacteria
NTUH: National Taiwan University Hospital
OR: Odds ratio
TB: Tuberculosis
